# A New Hæmostatic Means—Sheep Brains

**Published:** 1846-02

**Authors:** 


*A new hæmostatic means—sheep brains.*—M. Dupuy, at the sitting of the Academy of Medicine in Paris, 17th June, stated, that the cerebral matter of sheep possessed in a very high degree the property of coagulating the blood, and of immediately arresting hœmorrhages. A small portion of the brain injected into the femoral vein of an animal produced death in a few minutes. The blood was found coagulated in the heart, and in the vessels, as M. D. had predicted. He thought that surgeons might profit by this fact.—*Jour des Con. Médico-Chir.—Ibid.*

